# Impact of Sleep and Activity on Glycemic Control and Quality of Life in Haitian Children and Youth with Type 1 Diabetes

**DOI:** 10.1155/2023/4289288

**Published:** 2023-05-05

**Authors:** Melanie Babinski, Regina Duperval, Ketly Altenor, Julia von Oettingen

**Affiliations:** ^1^Department of Pediatrics, Montreal Children's Hospital, Montreal, QC, Canada; ^2^Kay Mackenson Clinic, Port-au-Prince, Haiti; ^3^Department of Pediatrics, Division of Nephrology, Montreal Children's Hospital, Montreal, QC, Canada; ^4^Department of Pediatrics, Division of Endocrinology, Montreal Children's Hospital, Montreal, QC, Canada; ^5^Research Institute of the McGill University Health Centre, Montreal, QC, Canada

## Abstract

**Background:**

Sleep and physical activity affect overall health. In youth with type 1 diabetes (T1DM), they may improve glycemic control. Data from low-income countries are lacking.

**Objective:**

To describe sleep and activity in Haitian children and youth with T1DM, and examine their impact on glycemic control, health-related quality of life (HRQL), and life satisfaction (LS).

**Methods:**

This cross-sectional study in Haiti included people with T1DM aged 8–25 years. Wristbands (Mi Band 3) tracked activity (step count and activity time) and sleep (sleep duration, light sleep, and deep sleep). The Diabetes Quality of Life in Youth (DQOLY) questionnaire was used to evaluate HRQL and LS. Point-of-care (POC) hemoglobin A1c values were recorded. Linear regression was used to assess the relationship between sleep, activity, HbA1c, HRQL, and LS.

**Results:**

We included 66 participants (59% female, mean age 17.8 ± 4.8 years, mean diabetes duration 3.7 ± 3.4 years, and mean BMI *Z*-score −0.86 ± 1.1). Mean HRQL was 63/100, and mean LS was 65/100. Mean HbA1c was 11.3%. Maximum HbA1c measure was 14% on the POC machine, and 23 participants (35%) had HbA1c recorded as 14%. Mean daily step count was 7,508 ± 3,087, and mean sleep duration was 7 h31 ± 1 h17. When excluding participants with HbA1c ≥ 14%, shorter sleep duration was significantly associated with higher HbA1c (*p* = 0.024). Sleep duration and step count were not associated with HRQL or LS.

**Conclusions:**

Children and youth with T1DM in Haiti have poor glycemic control and low HRQL and LS. Their sleep and activity habits are similar to peers. While activity did not affect HbA1c, HRQL, or LS, shorter sleep duration was associated with higher HbA1c in participants with HbA1c < 14%. Prospective studies with larger sample sizes are needed to validate our findings.

## 1. Introduction

Type 1 diabetes mellitus (T1DM) is one of the most common chronic diseases of childhood, affecting over one million youth under 20 years old across the globe, and on the rise each year [1]. Managing T1DM is a complex process that requires frequent self-monitoring and insulin administration. The International Society for Pediatric and Adolescent Diabetes (ISPAD) clinical guidelines [2] highlight the need for a structured approach to nutrition, activity, sleep, and mental health. In low- and middle-income countries (LMIC) such as the Republic of Haiti, reduced access to healthcare and lack of insulin are certainly significant barriers to optimal diabetes management. However, data suggest that glycemic control is poor even when proper follow-up and adequate care supplies are provided [3, 4], and the rates of microvascular complications are concerningly high [5]. Little research has been conducted in these countries regarding modifiable aspects of diabetes management such as physical activity and sleep.

Physical activity and adequate sleep are two lifestyle factors known to be important for overall health in the general population for various reasons, ranging from cardiovascular, metabolic, and bone health, as well as improved cognition and mental health [6].

The benefits of activity in children and youth with T1DM are also similar to the general population, as demonstrated by a recent systematic review in 2019 that found positive effects on lipid profile, physical fitness, quality of life, body size, and body composition [7]. However, the effect of physical activity on glycemic control is less clear. One systematic review and meta-analysis in youth and adults found that supervised moderate-to-high intensity exercise conferred no overall benefit on hemoglobin A1c (HbA1c) [8]. However, others that only included those under 18 have shown that physical activity interventions significantly reduced HbA1c [9]. A few recent meta-analyses suggest that the effect on HbA1c is greatest with more frequent, longer [10], and higher intensity [11] physical activity.

In terms of sleep, the National Sleep Foundation recommends that teenagers get 8 to 10 hours of sleep per night, and young adults and adults get 7 to 9 hours [12]. Research has shown that individuals with T1DM have shorter sleep time and poorer sleep quality compared to controls [13]. Shorter sleep, worse sleep quality, and increased sleep variability have all in turn been linked to suboptimal glycemic control both in adults [14] and children [15] in two recent systematic reviews.

There is a paucity of data on sleep and activity habits within the Haitian population in general, much less in those with T1DM. In this observational study, our aim was to describe the sleep and activity habits of children and youth with T1DM in Haiti and to determine if these were significantly associated with glycemic control. We also aimed to assess health-related quality of life (HRQL) in this population and examine its association with sleep and activity. This is part of a larger study—the DÉterminants Sociaux et Individuels de santé en Diabète pÉdiatrique (Social and individual determinants of pediatric diabetes, DESIDE)—conducted at the Kay Mackenson Clinic in Haiti. DESIDE aims to better understand how cultural, socioeconomic, psychosocial, activity-related, and diet-related factors affect children and youth living with diabetes in Haiti.

## 2. Methods

### 2.1. Study Design and Participants

This was a single-center, cross-sectional study conducted at the Kay Mackenson Clinic (KMC), a clinic for children with chronic, noncommunicable diseases in Montrouis, Haiti. It is the second largest diabetes clinic in Haiti. Participants were eligible if they were between the ages of 0 to 25 years at diagnosis of diabetes, they resided in Haiti, and they had at least one parent of Haitian ancestry. Exclusion criteria were an inability to provide informed consent (by participants themselves if 18 and over or by parent/legal guardian if younger than 18 years) and/or refusal of a home visit by the study team.

### 2.2. Study Procedures and Data Collection

Participants were visited in their homes by the research team (nurse, social worker, and research assistant) for one study visit where data were collected, questionnaires administered, and actigraph wristbands distributed. Demographic data (date of birth, sex, date of diagnosis, home address, living situation, schooling, and employment) were collected as part of the study questionnaire. Socioeconomic status (SES) was assessed using the Demographic and Health Survey household questionnaire for low-income countries [16]. From this survey, an SES score was computed. Details regarding the survey and methodology have previously been reported by the DESIDE group [17]. Clinical data related to diabetes were collected from health records, including insulin regimen and total daily insulin dose, as well as other past medical history and other medications. Weights and heights were obtained using a SECA mechanical scale and SECA portable stadiometers (SECA North America, CA, USA). BMI was calculated using the standard formula (weight in kilograms divided by height in meters squared), and BMI *Z*-scores were calculated using the Canadian Pediatric Endocrine Group “Shiny” WHO anthropometric *Z*-scores calculator [18].

The Siemens DCA Vantage (Siemens Medical Solutions USA, Inc., PA, USA) point-of-care instrument was used to measure participants' HbA1c levels. Of note, the maximum measure on this instrument is 14% (129.5 mmol/mol).

### 2.3. Activity and Sleep Tracking

Participants wore the actigraph wristbands Mi Band 3 (MB, Xiaomi Communications Co., Ltd., Beijing, China—https://www.mi.com/in/mi-band-3/). They were instructed to wear the wristbands on their nondominant wrist for at least 7 days and nights. After receiving them during the study visit, they returned the wristbands at their next clinic visit. Accelerometry data were downloaded from the MB using proprietary software onto a study computer and transcribed into a spreadsheet for data analysis.

MB tracks activity and sleep by use of a 3-axis accelerometer and heart rate monitor. In terms of sleep, the accelerometer detects body movements and begins to measure sleep when there has been no movement for a certain amount of time. The exact timeframe is not made available by the company, but other consumer actigraphs use 1 hour of little to no movement as the start of the sleep period [19]. When sufficient movement is detected, wake-up time is registered. Total sleep time is then calculated as the time from sleep onset to wake-up time, minus any periods of wakefulness.

MB differentiates between light sleep and deep sleep by combining data about body movement and heart rate, such that less movement and lower heart rate correspond to deep sleep. However, specific thresholds for differentiation are not made available by the manufacturer [19, 20].

Though wristband actigraphy is not as reliable as the gold standard of polysomnography (PSG), there is one study demonstrating that MB has reasonable accuracy in measuring sleep duration when compared to a wristband with manual stop-start measurement for sleep [21] and another demonstrating strong correlation with subjective sleep reports [20]. Only one study [22] has compared MB with PSG and found that MB correlated well with PSG for the measurement of “time in bed,” though did overestimate total sleep time by about 70 minutes.

We also calculated sleep variability for each subject by using the standard deviation of their daily total sleep duration.

MB also measures various physical activity parameters. As with sleep, specific methods for measuring activity metrics are not made available by the manufacturer. However, information can be gleaned by comparing to other wristbands and looking at validation studies. Activity metrics measured by MB include:

1. Daily steps: measured using the accelerometer
2. Activity time: MB sets a goal of 30 minutes “active time” per day, which has led other authors to postulate that it is measuring moderate-vigorous physical activity (MVPA) using the accelerometer and heart rate monitor, given that this is similar to MVPA recommendations for adults [23].

Step count has been shown to have the highest validity when compared to other activity metrics measured by consumer-wearable activity trackers [24]. Multiple recent studies have found that MB has a high validity for step count when compared with a gold-standard actigraph [20, 23, 25]. Conversely, activity time is weakly correlated with gold standard measures [23].

### 2.4. Health-Related Quality of Life

The Diabetes Quality of Life in Youth (DQOLY) questionnaire is a 52-item instrument used to assess health-related quality of life (HRQL) in adolescents and young adults with type 1 diabetes. It is divided into 3 subscales: disease impact (26 items), disease-related worries (11 items), life satisfaction (17 items), plus a 52^nd^ question used to assess overall health perception [26]. However, the full questionnaire has been shown to have poor construct validity and is lengthy to administer. A short form 21-item DQOLY (DQOLY-SF) was developed in 2006 by Skinner et al., and it demonstrated improved construct validity [27]. A culturally adapted Creole version of the DQOLY-SF was created as part of the DESIDE study and was shown to have adequate internal consistency and validity in this same population of Haitian children and youth with T1DM [17]. As well, the life satisfaction subscale was validated and found to be distinct from the other components of HRQL.

In our study, the Creole DQOLY-SF and life satisfaction subscale were used to evaluate health-related quality of life and life satisfaction, respectively. Both the DQOLY-SF and life satisfaction scores were normalized on a scale of 0 to 100.

### 2.5. Outcomes

The primary outcomes of this study were sleep and activity habits among children and youth living with type 1 diabetes in Haiti and their association with glycemic control (as defined by HbA1c). Specifically, we measured total sleep duration, light and deep sleep, daily step count, and daily activity time.

The secondary outcome was the association of sleep and activity with HRQL and LS.

We did not examine the relationship between HRQL/LS and glycemic control, as a previous study, also part of the DESIDE protocol, had shown no significant correlation in this population cohort [17].

### 2.6. Data Analysis

Descriptive statistics were used for participant baseline characteristics. The mean and standard deviation, as well as median and interquartile range, were calculated as appropriate for total sleep duration, light sleep, deep sleep, step count, and activity time.

Linear regression was used to evaluate sleep and activity as predictors of HbA1c, HRQL, and LS.

Given that the agreement between PSG and MB for sleep stages (light vs. deep) was shown to be quite low in one study [22], as well as the fact that there are no official recommendations for light and deep sleep durations in children and adolescents, we chose to use only total sleep duration as the sleep parameter in the regression analysis.

We used daily step count as the only activity parameter in our regression analysis. This is first because, as discussed previously, it has been validated against gold-standard measures while other metrics have not been. As well, step count is the most widely used activity metric across other literature. In multivariate regression models, the two independent variables, sleep and activity, were adjusted for age, sex, diabetes duration, BMI Z-score, and SES score.

A significant subset of participants (35%, *n* = 23/66) had HbA1c measured as 14%, which was the maximum measure on the point-of-care machine, and their true HbA1c may have been higher than 14%. Given the potential for this to create a ceiling effect masking correlations, analyses were run both including and excluding this subset of participants.

Analyses were run using SAS 9.3 (SAS Institute Inc.). A *p* value of <0.05 was considered significant.

## 3. Results

### 3.1. Study Participants and Baseline Characteristics

The study included a total of 66 participants. 39 participants (59%) were female, and the mean age was 17.8 ± 4.8 years. All participants were of Haitian ethnicity. Their mean diabetes duration was 3.7 ± 3.4 years, and their mean BMI *Z*-score was −0.86 ± 1.1. Their mean HbA1c was 11.3 ± 2.6% (100 ± 5 mmol/mol), and 23 participants (35%) had HbA1c ≥ 14% (129.5 mmol/mol). The mean socioeconomic score was 9.1 out of 15. All participants were attending school, and 4/24 participants (17%) above 18 years of age were employed. Baseline characteristics are shown in Table 1.

### 3.2. Sleep Data

The summary of all sleep data is shown in Table 2. The average number of nights recorded per participant was 8 nights (range 3–23 nights), and there were 6 participants who had 5 nights or less recorded.

The mean total sleep duration was 7 h31 ± 1 h17 per 24 hours, with a median of 7 h29 (IQR 6 h59, 8 h14). Descriptive statistics were recalculated excluding participants with 5 nights or less recorded, but they did not significantly change (data not shown). The mean light sleep was 5 h53 ± 1 h09, with a median of 6 h03 (IQR 5 h04, 6 h40). The mean deep sleep was 1 h38 ± 0 h39, with a median of 1 h33 (IQR 1 h16, 2 h03). The mean sleep variability was 1 h18 ± 0 h52, with a median of 1 h05 (IQR 0 h40, 1 h35). Data were not collected regarding sleep duration on weeknights versus weekends.

### 3.3. Activity Data

The summary of all activity data is shown in Table 3. The average number of days recorded per participant for activity data was 10 days (range 3–25 days), with only 2 participants having 5 days or less recorded.

The mean daily step count was 9,448 ± 8,431 steps per 24 hours, with a median of 7,910 steps (IQR 5,365, 10,592). There were 4 outliers who had mean daily steps greater than 30,000, which was likely a measurement error rather than true variation in the sample. When these were excluded, the mean step count was 7,508 ± 3,087 steps, with a median of 7,785 (IQR 5,125, 10,167). Regression analyses were conducted both including and excluding these outliers, but this did not significantly change the results (data not shown).

The mean daily activity time was 123 ± 97 minutes, with a median of 104 minutes (IQR 77, 132). The same 4 outliers had mean daily activity time >350 minutes, and when these were excluded, the mean was 101 ± 38 minutes, with a median of 101 minutes (IQR 75, 128).

### 3.4. HRQL Data

The mean HRQL score was 63 ± 15 (out of 100) with a median of 64 (IQR 52, 74). The mean LS subset score was 65 ± 23 (out of 100) with a median of 68 (IQR 52, 83).

### 3.5. Associations with HbA1c

In univariate regression analyses, higher HbA1c was associated with younger age (*p* = 0.005) and lower BMI *Z*-score (*p* = 0.0016) but not with sex, diabetes duration, or SES score nor with daily step count or sleep duration. However, 35% (*n* = 23/66) of participants had an HbA1c of 14%, which could have truthfully been higher due to the point-of-care machine's inability to read values greater than 14%. Given that this could have caused a ceiling effect masking certain predictors, we reanalyzed the data excluding participants with HbA1c greater than or equal to 14%. In the remaining 43 participants, shorter sleep duration was not associated with HbA1c (*p* = 0.292) in univariate regression, but became significantly associated with higher HbA1c in a multivariate model adjusted for age, sex, BMI *Z*-score, and diabetes duration (*p* = 0.024). The association between sleep variability and HbA1c approached significance in this subset of participants with HbA1c < 14% (*p* = 0.056), but this association was no longer significant (*p* = 0.71) in multivariate regression adjusted for age, sex, BMI *Z*-score, and diabetes duration.

Step count was not correlated with HbA1c (*p*=0.88 and *p*=0.63 in univariate and multivariate analyses, respectively). Even when we excluded participants with HbA1c ≥ 14%, there was no significant association (*p*=0.65). See details in Tables 4 and 5.

### 3.6. Associations with HRQL and Life Satisfaction

Better HRQL was associated with younger age in univariate regression (*p*=0.029) and multivariate regression (*p*=0.03) adjusted for sex, BMI *Z*-score, and diabetes duration.

Better HRQL was also marginally correlated with male sex in univariate regression (*p*=0.067) and significantly correlated in multivariate regression (*p*=0.04) adjusted for age, BMI *Z*-score, diabetes duration, and SES score. HRQL was not associated with BMI *Z*-score, diabetes duration, or SES score. HRQL was not associated with sleep duration or step count in univariate or multivariate regression.

Better life satisfaction correlated with younger age (*p*=0.043) in univariate regression, as well as with a higher SES score in univariate (*p*=0.023) and multivariate regression adjusted for age, sex, BMI *Z*-score, and diabetes duration (*p*=0.0097). Neither sex nor BMI *Z*-score were significantly correlated with life satisfaction. In univariate and multivariate models adjusted for age, sex, BMI *Z*-score, diabetes duration, and SES score, life satisfaction was not associated with step count nor with sleep duration.

Given the large age range of our cohort and keeping in mind that sleep and activity habits differ greatly across age groups, we analyzed our data separately for children (<13 years old) and adolescents and young adults (≥13 years old). The findings were no different for descriptive measures nor for regression analyses (data not shown).

## 4. Discussion

In this observational single-center study, we found that Haitian children and youth with diabetes have sleep and activity habits that are similar to peers in high-income countries. They concurrently have overall suboptimal glycemic control, low HRQL, and low LS. In secondary analyses after exclusion of 23 participants (35%) with HbA1c ≥ 14%, shorter sleep duration was associated with higher HbA1c among remaining participants with HbA1c < 14%. Sleep variability was not correlated with glycemic control. Activity levels had no association with glycemic control. Neither sleep nor activity was associated with better quality of life.

### 4.1. Glycemic Control

The mean HbA1c of 11.3% (100 mmol/mol) in this sample is significantly higher than similarly aged cohorts in high-income countries. While in other resource-limited settings, HbA1c values are somewhat similar [28–30]; in most European countries, the mean HbA1c ranges between 7.5 and 8.5% (58–69 mmol/mol) [31, 32], and in the United Kingdom and United States, it rests between 8 and 9% (64–75 mmol/mol) [31, 33]. ISPAD recommends a target HbA1c of <7.5% for those who lack access to continuous glucose monitoring and more advanced insulin delivery technology [34]. However, in our cohort, only 5 of 66 participants (7.5%) had HbA1c < 7.5%. The reasons for suboptimal glycemic control among this population are multifactorial and likely strongly mediated by uniformly unfavorable social determinants such as parental educational level [35] and health literacy [36], as well as urban versus rural residence [28]. Our study investigated the potential impact of the two lifestyle factors, physical activity and sleep, and found that only shorter sleep duration was significantly associated with glycemic control.

### 4.2. Physical Activity

In this study, we assessed physical activity using wristband accelerometers and used daily step count as our main activity metric. The most widely used recommendations for daily step count from Tudor-Locke et al. state that adolescents 12–19 years old should get >10,000 steps/day, while adults 20–65 years old should get >7,000 steps/day [37, 38]. The average step count in our sample was 7,508 steps/day (when excluding outliers), which is just above the recommended amount for adults. Children and youth with T1DM had between 8,000 and 9,000 daily steps in two recent studies conducted in Canada [39] and Poland [40]. The mean daily step count within our cohort is therefore overall similar or slightly lower than that reported in peers with T1DM and the general population in higher income countries.

We did not find an association between activity (specifically, step count) and glycemic control, which is consistent with other literature [7, 8]. In a recent systematic review assessing the benefits of physical activity in children and youth with type 1 diabetes [7], Absil et al. found that although exercise confers multiple benefits to overall health (physical fitness, blood lipid profile, body size, and composition), the positive effect on glycemic control is not as widely supported. This lack of association is likely due, in part, to a wide variation in study protocols and insufficient sample sizes. It is not surprising, therefore, that our observational study with a relatively small sample size did not show a significant association between activity and HbA1c.

### 4.3. Sleep

In this study, we assessed sleep using the total sleep time as measured by wristband actigraphy. The mean sleep duration in our cohort was 7 h31 min, which meets the National Sleep Foundation (NSF) recommendations for sleep duration in young adults of 7–9 hours per night [12]. A recent systematic review found that the average sleep duration among US adolescents with T1DM was between 6.3 and 8.6 h [15], suggesting that our sample is similar to others. The mean sleep variability in our cohort was 1 h18 min, which is also similar to two recent T1DM adolescent cohorts in the United States [41–43].

We found that shorter sleep duration was associated with a higher HbA1c in participants with an HbA1c < 14%. While previous literature has linked shorter sleep to suboptimal glycemic control in young adults with T1DM [13, 44], the effect is less clear among children and adolescents. Some studies have shown no association [43, 45], while others have found a correlation between shorter sleep and higher HbA1c [42, 46], including one RCT that demonstrated improved glycemia with a sleep extension intervention [47].

Shorter sleep duration has been associated with less frequent self-monitoring of blood glucose in several studies [42, 48, 49], suggesting that part of the impact of sleep on glycemic control may be indirect, by impairing executive function and making diabetes self-management more difficult.

From a physiological perspective, sleep deprivation has been shown to promote insulin resistance in both healthy subjects [50] and those with type 1 diabetes [51], and both lower sleep efficiency and quality also seem to play a role by decreasing insulin sensitivity [52] and increasing glucose variability [53].

It is important to consider that the association between sleep and glycemic control is likely bidirectional [46, 49, 54]. While shorter, poorer sleep may lead to deteriorating glycemic control through various mechanisms, hyperglycemia may also lead to poor sleep by way of increased nocturnal voiding, thirst, and need for overnight insulin injections. As elaborated on by Barone and Menna-Barreto [55], rapid glycemic changes may lead to sympathetic nervous system activation that fragments sleep, and suboptimal glycemic control may play a role in the development of central apnea and poorer quality sleep.

### 4.4. Limitations

Our study has a few limitations. First, the sample size was relatively small, although it is the largest sample of children and youth with type 1 diabetes in Haiti to our knowledge. As well, it was cross-sectional in design, which limits the ability to determine causation.

Moreover, there are some limitations to the use of a consumer-wearable wristband such as MB for measuring sleep and activity. MB uses movement and heart rate to estimate bedtime and wake time, which is not as precise as the gold standard of polysomnography and has been shown to overestimate the total sleep duration in at least one study [22]. MB is not able to calculate more advanced sleep parameters such as sleep onset latency and sleep efficiency, which would be helpful for offering a more complete assessment of participants' sleep. As well, we were not able to differentiate between weeknight and weekend sleep patterns (social jetlag) when looking at sleep variability, which could have affected glycemic control. We did not assess participants' sleep quality.

For physical activity, MB relies on the accelerometer to estimate all activity parameters. Since it is worn on the wrist, it is possible that some outliers had an overestimation of activity due to manual labor that required repetitive, large hand motions that could be mistaken for steps.

Another limitation was the lack of access to continuous glucose monitoring. Our ability to capture glycemic patterns was thus limited to one point-of-care HbA1c test. As such, glycemic patterns at the time of sleep and activity measurement could have differed from what was reflected in the HbA1c.

## 5. Conclusion

Our findings are an important addition to the scant body of the literature surrounding young people with chronic diseases in low and middle-income countries. We documented normal physical activity and sleep patterns contrasting with suboptimal glycemic control and low HRQL in our cohort. Physical activity levels were unrelated to both glycemic control and HRQL, while shorter sleep was associated with a higher HbA1c. This emphasizes the importance of evaluating sleep habits at all healthcare visits for people living with type 1 diabetes and encouraging healthy sleep hygiene. Our study highlights the need for further research examining sleep and glycemic control, particularly among children and youth in lower-income countries.

## Figures and Tables

**Table 1:** Baseline characteristics.

Variables	*N* (%)
	Mean ± SD
Female sex	39 (59)
*Ethnicity*	
Haitian	66 (100)
Other	0 (0)
Age (yrs)	17.8 ± 4.8
Diabetes duration (yrs)	3.7 ± 3.4
BMI *Z*-score	−0.86 ± 1.1
Hemoglobin A1c (%)	11.3 ± 2.6
Participants with HbA1c ≥ 14%	23 (35)
HRQL (out of 100) (*n* = 43)	63 ± 15
LS (out of 100) (*n* = 43)	65 ± 23
SES score (out of 15) (*n* = 56)	9.1 ± 3.1
School attendance	65 (98)
Employment in those ≥18 yo (*n* = 24)	4 (17)
*Parental education level (mother/father) (n* *=* *59)*	
None	12 (21)/9 (15)
Elementary school	22 (38)/18 (31)
High school	6 (10)/15 (25)
Unknown	18 (31)/17 (29)

SD = standard deviation; BMI = body mass index; HRQL = health-related quality of life; LS = life satisfaction.

**Table 2:** Sleep data (*N* = 66).

Variables	Mean	SD	Median	*Q*1	*Q*3
Sleep duration (h : mm)	7 : 31	1 : 17	7 : 29	6 : 59	8 : 14
Light sleep	5 : 53	1 : 09	6 : 03	5 : 04	6 : 40
Deep sleep	1 : 38	0 : 39	1 : 33	1 : 16	2 : 03
Sleep variability	1 : 18	0 : 52	1 : 05	0 : 40	1 : 35

**Table 3:** Activity data (*N* = 62).

Variables	Mean	SD	Median	*Q*1	*Q*3
Daily steps	7,508	3,087	7,785	5,125	10,167
Daily activity time (mins)	101	38	101	75	128

**Table 4:** Univariate predictors of HbA1c, HRQL, and LS.

Variables	*HbA1c*		*HRQL*		*LS*	
	*β*-Estimate	*P* value	*β*-Estimate	*P* value	*β*-Estimate	*P* value
Age (yrs)	−0.18	**0.0051**	−0.83	**0.029**	−1.21	**0.043**
Female sex	−0.69	0.29	−6.79	**0.067**	−0.59	0.92
Diabetes duration (yrs)	−0.11	0.23	−0.71	0.2	−1.52	**0.071**
BMI *Z*-score	−0.9	**0.0016**	−1.49	0.38	1.93	0.47
SES score	−0.0086	0.55	0.51	0.42	2.26	**0.023**
Step count (1,000 steps)^†^	0.016	0.88	0.57	0.38	0.15	0.88
Sleep duration (mins)	−0.0052	0.22	0.0045	0.85	0.02	0.6

^†^Regression was conducted using “daily step count/1,000” to express step count in increments of 1,000 steps rather than single steps, for ease of presenting *β*-estimate. The bold values are those with *p* < 0.05 or close to 0.05.

**Table 5:** Multivariate predictors of HbA1c, HRQL, and LS (adjusting for age, sex, diabetes duration, and BMI *z*-score).

Variables	*HbA1c (including values* *≥* *14%)*		*HbA1c (excluding values* *≥* *14%)*		HRQL		*LS*	
	*β*-Estimate	*P* value	*β*-Estimate	*P* value	*β*-Estimate	*P*value	*β*-Estimate	*P* value
Sleep duration (minutes)	−0.005	0.22	−0.009	**0.024**	0.0052	0.82	0.026	0.5
Step count (increments of 1,000 steps)^*∗*^	−0.018	0.63	0.015	0.65	−0.044	0.84	−0.33	0.32

The bold values are those with *p* value  <0.05.

## Data Availability

The data used to support the findings of this study are available from the corresponding author upon reasonable request.
